# Spectroellipsometric characterization and modeling of plasmonic diamond-like carbon nanocomposite films with embedded Ag nanoparticles

**DOI:** 10.1186/s11671-015-0854-y

**Published:** 2015-04-01

**Authors:** Iryna Yaremchuk, Šarunas Meškinis, Volodymyr Fitio, Yaroslav Bobitski, Kestutis Šlapikas, Arvydas Čiegis, Zigmas Balevičius, Algirdas Selskis, Sigitas Tamulevičius

**Affiliations:** Department of Photonics, Lviv Polytechnic National University, S. Bandera Str. 12, Lviv, 79013 Ukraine; Institute of Materials Science, Kaunas University of Technology, Savanoriu Av. 271, Kaunas, LT-50131 Lithuania; Institute of Technology, University of Rzeszow, T. Rejtana Str. 16b, Rzeszow, 35959 Poland; Laboratory of NanoBioTechnology, Center for Physical Sciences and Technology, Goštauto str. 9, Vilnius, LT-01108 Lithuania; Institute of Chemistry, Center for Physical Sciences and Technology, Goštauto str. 9, Vilnius, LT-01108 Lithuania

**Keywords:** DLC:Ag nanocomposite, Surface plasmon resonance, Effective medium theory

## Abstract

Diamond-like carbon nanocomposite films with embedded silver nanoparticles are considered experimentally (spectroellipsometric characterization) and theoretically (modeling of optical properties). Metallic nanocomposite films were synthesized by reactive magnetron sputtering and were studied by transmission electron microscope (TEM) and atomic force microscope (AFM). The optical constants of the films were determined from spectroscopic ellipsometry measurements and were modeled using the Maxwell-Garnett approximations. Comparison between the extended and renormalized Maxwell-Garnett theory was conducted. Surface plasmon resonance peak have been found to be strongly dependent on the shape of nanoparticles and interaction between them.

## Background

Metal-doped diamond-like carbon (DLC) films have potential applications in many practical applications and for theoretical studies of various physical phenomena, which make them compatible to use in large variety of applications. They demonstrate superior toughness, thermal stability, excellent tribological properties, as well as relatively lower residual stresses than that of pure diamond-like carbon films [1-4]. Numerous metallic components (Ti, W, Ag, Cu, Au, etc.) have been used to modify structures and properties of the films [5-8]. Among them, the Ag-incorporated diamond-like carbon films have increasingly gained attention because of wide applications in optical device applications [9], biomedical implants due to surface anti-bacterial properties [10], solar energy [11], electronic devices [12], for catalysis effect [13], and tribological applications [14,15]. When atomic concentration of the group IB metal (silver, copper, or gold) in the diamond-like carbon film is more than several atomic percents, due to silver (copper, gold) segregation, silver (copper, gold) nanoclusters embedded in the diamond-like carbon matrix were detected [5,8,9]. Therefore, in these diamond-like nanocomposite films, surface plasmon resonance was observed [8,9,16].

In the field of photonics, nanocomposite materials containing nanoscale particles of noble metals are of great interest because of their unique optical characteristics originating from the strong interaction between incident light and metallic nanoparticles [17,18]. This interaction results in collective oscillations of electron clouds, called surface plasmon resonance (SPR), at the interface of the metallic nanoparticles and the dielectric matrix. The resonance frequency of this interaction is strongly dependent on the metal, the surrounding dielectric medium, as well as the size and shape distribution of the nanoparticles [19,20]. It must be noted that silver nanoparticles exhibit a sharp and distinct optical response (SPR) in the visible region of electromagnetic spectrum, which is extremely important for optoelectronic applications. In addition, it should be mentioned that plasmonic nanocomposites have some advantages over nanoparticles such as increased environmental stability as well as additional possibilities of tuning of the optical properties.

Thus, for a given metal, there are several means for tuning the SPR spectral properties, namely, dielectric constant of the host, nanoparticles shape, nanoparticles concentration, and nanoparticles size. The present research focuses on detailed study of these possibilities both experimentally, using magnetron-sputtered Ag-DLC thin films (spectroellipsometric characterization), and theoretically (modeling of optical properties).

## Methods

The DLC as well as DLC-based silver nanocomposite films were deposited employing the reactive direct current (DC) magnetron sputtering of the silver target. The diameter of magnetron was 3 in. Monocrystalline silicon and quartz substrates were used. Mixture of the hydrocarbon (acetylene) and argon gas was applied. Substrate-target gap was 10 cm, base pressure 5 × 10^−4^ Pa, and work pressure (4 ± 1) × 10^−1^ Pa. During the deposition, sample was grounded or 50 V negative substrate bias was used. During the process, the deposition rate was kept constant monitoring a quartz crystal microbalance sensor and manually adjusting the magnetron current. Thickness of the films was approximately 100 nm.

DLC:Ag film deposited on the grounded substrate contained 22 at.% of silver and DLC:Ag film deposited by using −50 V substrate bias contained 8 at.% of silver. The composition was defined by using X-ray photoelectron spectroscope KRATOS ANALYTICAL XSAM800 (Kratos Analytical Inc., Spring Valley, NY, USA).

Transmission electron microscope (TEM) FEI Tecnai G2 F20 X-TWIN (FEI, Hillsboro, OR, USA) equipped with an energy dispersive X-ray spectroscope (EDS) as well as atomic force microscope (AFM) NanoWizard®3 (JPK, Berlin, Germany) were used for evaluation of the dimensions of the silver nanoclusters. Particle size distribution analysis was done by measuring diameters of the bright spots in AFM image. ImageJ software was applied.

Refractive index and extinction coefficient dispersion curves of the films were obtained employing J.A. Woollam RC2 spectroscopic ellipsometer (J.A. Woollam Co., Lincoln, NE, USA) with two rotating compensators. Ellipsometric measurements have been carried out in a spectral range from 210 to 1700 nm and angle of incidence of 75°. The experimental ellipsometric data were analyzed by means of the J.A. Woollam program CompleteEase (J.A. Woollam Co., Lincoln, NE, USA).

Optical absorbance and reflectance spectra of the films were measured by using an optical spectrometer Avantes (Avantes BV, Apeldoorn, The Netherlands) that is composed of a deuterium halogen light source (AvaLight DHc) and spectrometer (Avaspec-2048). The absorbance of the films was analyzed in the wavelength region from 180 to 1,100 nm.

## Results and discussion

**Figure 1:**
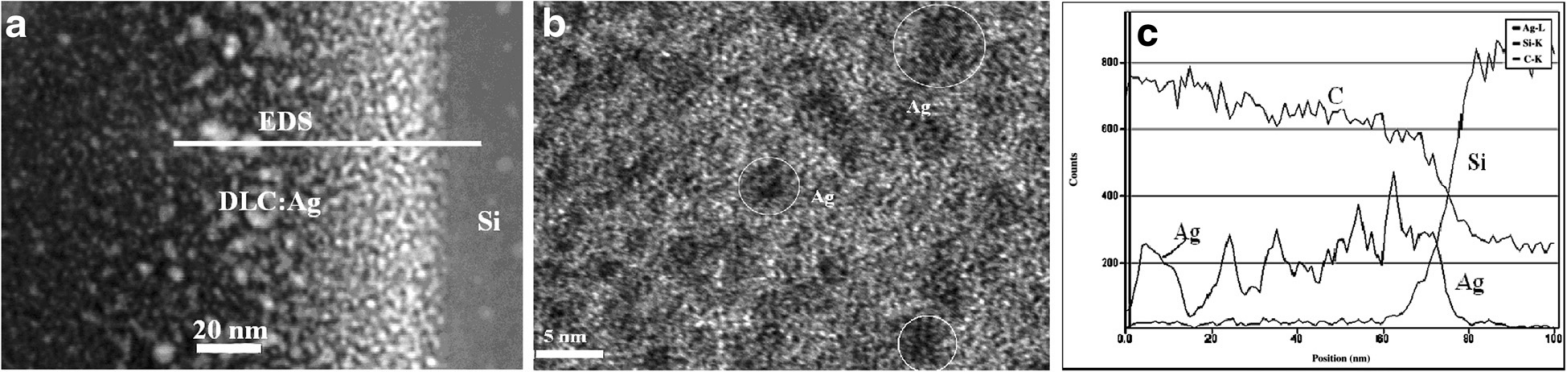
**TEM image (overall view). (a)** High-resolution TEM image and **(b)** EDS profiling across the line shown in **(a)** of **(c)** DLC: Ag films containing 22 at.% of silver.

The structure of DLC:Ag nanocomposite thin films was studied by TEM and AFM. Silver nanoclusters of 5 to 10 nm size can be seen in high-resolution TEM films cross-section photo (Figure 1a,b).

**Figure 2:**
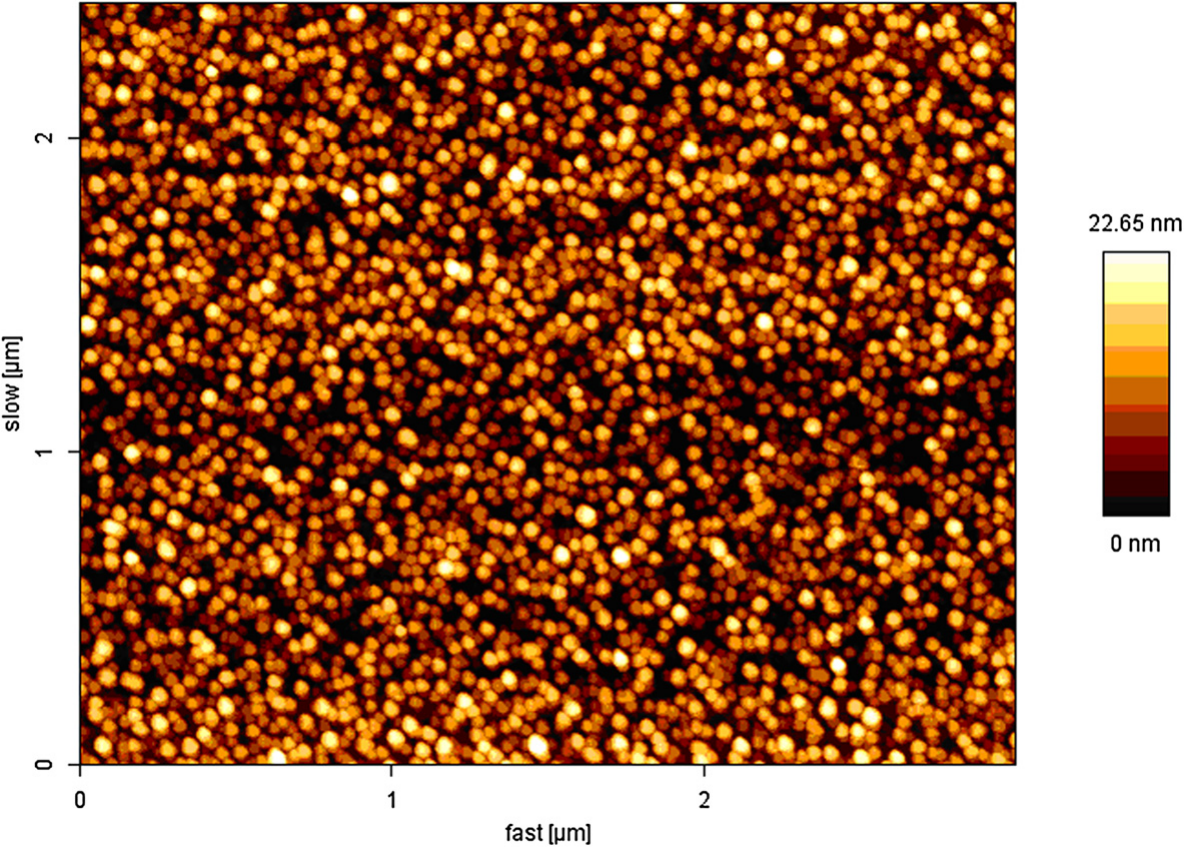
**Typical AFM image of the surface of DLC:Ag film (film containing 22 at.% of Ag).**

**Figure 3:**
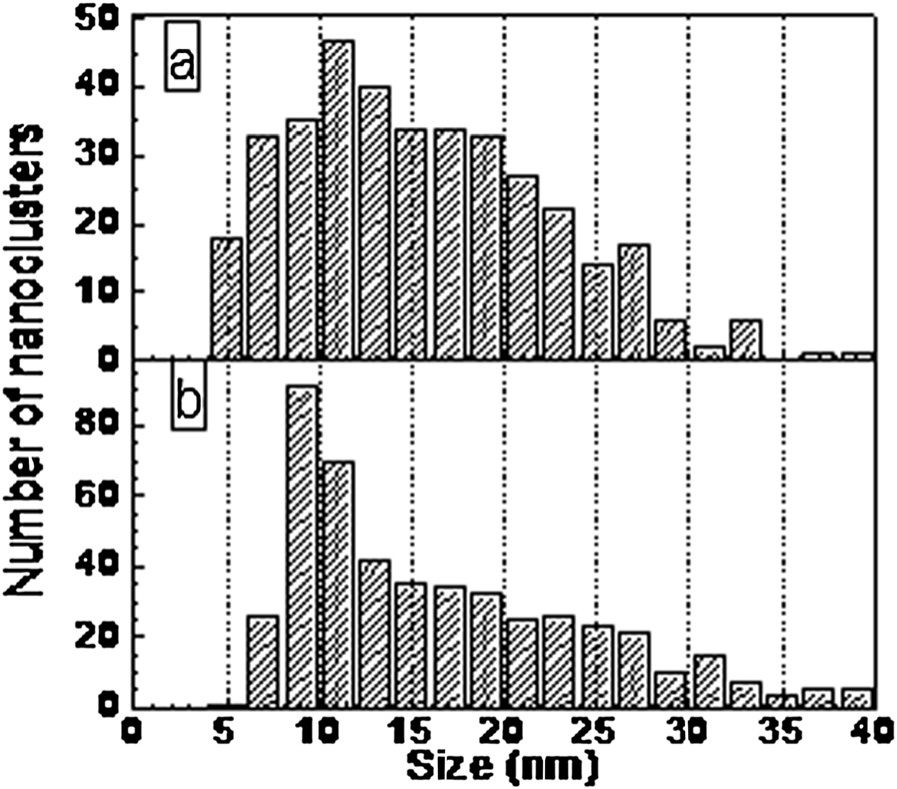
**Nanoparticle size distribution.** DLC:Ag films containing 22 **(a)** and 8 at.% **(b)** of Ag. Histograms were calculated by using AFM images.

According to the EDS, line profile size of silver nanoclusters is in 7- to 16-nm range (see Figure 1b). Silver nanoclusters even more clearly can be seen in AFM image of DLC:Ag film surface as bright circular spots (see Figure 2). Distribution of the diameters of silver nanoclusters calculated from the AFM image is presented in Figure 3. It can be seen in Figure 3 that the diameter of the silver nanoclusters calculated from the AFM image is in good accordance with the size of nanoclusters revealed by TEM and EDS line profile.

According to the AFM image (Figure 3), the size of silver nanoclusters is in 5- to 40-nm range and the most frequent diameter of the nanocluster is 12 nm. Thus, HRTEM and EDS profiling as well as AFM data on Ag nanocluster size are in good accordance. It should be mentioned that in [16] Ag nanoclusters were observed both by TEM and AFM for DLC films containing silver. While in [21], Cu nanoclusters were observed both by SEM and AFM for DLC:Cu films deposited by reactive RF diode sputtering.

In order to have an appreciation of the resonant absorbance behavior of metallic nanoparticles with respect to the incident radiation as well as the shape and size of the nanoparticles, we theoretically analyzed our experimental results.

The straightforward way to calculate the dielectric (optical) response would be to sum all contributions to the electrical polarization of the whole sample, including retarded electrodynamic multipole interactions of neighboring particles and the size, shape, and interparticle distance distributions in the sample.

Effective-medium theory is a powerful tool for describing the composite media. The most widely used is the Maxwell-Garnett (MG) theory [22,23] which is derived from the Lorentz local field relation covered in many textbooks. MG theory only includes the size effect of independent polarisable particles. However, complex systems containing only single stabilized nanoparticles are of limited interest for technical and practical applications. Single nanoparticles are of prime interest for fundamental research, but in nature, instead of systems with single nanoparticles, we often meet systems with many particles. Therefore, it is needed to take into account the dipole-dipole interactions between the particles. Moreover, such a theory ceases to be reliable if the filling fraction tends to be large [24]. In works of [25,26], the renormalized MG (RMG) approximation is proposed since it considers a renormalized (by the interactions) polarizability of the particles and is useful for higher filling fraction.

According to the RMG effective medium theory, we can define an effective permittivity *ε*eff for a composite containing metal nanoparticles (with permittivity *ε*m) embedded in a host matrix (with permittivity *ε*h) as:

1 $$ \frac{\varepsilon_{\mathrm{eff}}-{\varepsilon}_{\mathrm{h}}}{\varepsilon_{\mathrm{eff}}+2{\varepsilon}_{\mathrm{h}}}=\frac{4\pi }{3}\frac{f}{V}{\alpha}^{*}, $$

where *f* is the filling fraction; *V* is the nanoparticles volume. This relation between *ε*eff and α^*^ now allows the Maxwell-Garnett equation to be extended to nonspherical particles.

Renormalized average polarizability (α^*^) is given by [25]:

2 $$ {\alpha}^{*}=\frac{2\overline{\alpha}}{\kappa}\left\{1-\frac{\sqrt{1-\kappa \left(1-\delta \right)}}{2}\left[\sqrt{1-\nu }+\frac{ \arcsin \left({\nu}^{1/2}\right)}{\nu^{1/2}}\right]\right\}; $$

where $\overline{\alpha}=1/3\left(2{\alpha}_{\perp }+{\alpha}_{\left|\right|}\right)$, $\kappa =f{\left(\overline{\alpha}/{R}^3\right)}^2$, and *ν* = 3*κδ*/(1 − *κ*(1 − *δ*)).

Parameter of anisotropy is:

3 $$ \delta =\left({\alpha}_{\perp }-{\alpha}_{\left|\right|}\right)/\left(2{\alpha}_{\perp }+{\alpha}_{\left|\right|}\right). $$

The polarizability tensor components *α*_⊥_ and *α*_||_ are given as follows:

4 $$ {\alpha}_{\perp }=\frac{\varepsilon_{\mathrm{m}}/{\varepsilon}_{\mathrm{h}}-1}{\left({\varepsilon}_{\mathrm{m}}/{\varepsilon}_{\mathrm{h}}-1\right){n}_{\perp }+1}\left(\frac{V}{4\pi}\right),\kern0.24em {\alpha}_{\left|\right|}=\frac{\varepsilon_{\mathrm{m}}/{\varepsilon}_{\mathrm{h}}-1}{\left({\varepsilon}_{\mathrm{m}}/{\varepsilon}_{\mathrm{h}}-1\right){n}_{\left|\right|}+1}\left(\frac{V}{4\pi}\right), $$

where *n*_⊥_ and *n*_||_ are the geometric factors called the depolarization coefficients. For a spheroid with eccentricity *e* (*e* < < 1):

5 $$ {n}_{\perp }=\frac{1}{3}\mp \frac{1}{15}{e}^2,\;{n}_{\left|\right|}=\frac{1}{3}\pm \frac{2}{15}{e}^2, $$

where two signs correspond to prolate or oblate shape.

The relationship between the optical absorption coefficient *α* and the effective dielectric constant *ε*eff is given by [27]:

6 $$ \alpha =\frac{2\sqrt{2}\pi }{\lambda }{\left[{\left({\varepsilon}_R^2+{\varepsilon}_I^2\right)}^{1/2}-{\varepsilon}_R\right]}^{1/2}, $$

where *ε*R and *ε*I are the real and imaginary parts the effective dielectric constant, respectively.

For the modeling purposes, the dielectric constant of the metal bulk must be modified to take in account the decrease of the electron mean free path in the small particles. This will produce an increment in the predicted halfband width of the absorption spectra from the surface plasmon resonance. The complex dielectric constant correction due to the dependence on the frequency *ω* and particle size *R* from the nanoparticles is given as [28]:

7 $$ \begin{array}{l}{\varepsilon}_{1\mathrm{particle}}\left(\lambda, R\right)={\varepsilon}_{1\mathrm{bulk}}\left(\lambda \right),\\ {}{\varepsilon}_{2\mathrm{particle}}\left(\lambda, R\right)={\varepsilon}_{2\mathrm{bulk}}\left(\lambda \right)+\eta \frac{\omega_p{\lambda}^3}{{\left(2\pi c\right)}^3}\frac{V_f}{R},\end{array} $$

where *ω*_*p*_ is the plasma frequency (1.38 × 10^16^*s*^−1^), *V*f is the Fermi velocity of the conduction electrons (1.4 × 10^6^ms^−1^), *c* is the speed of light, and *η* is a factor (between 0.6 and 1). Dielectric constants of bulk silver were used from the work of Johnson and Christy [29].

The refractive index and extinction coefficient of host matrix (DLC film) in our calculations were used from the work of [9]. The values of the refractive index and extinction coefficient were obtained by regression of the spectroscopic ellipsometry data and extrapolated by fifth-order polynomial equations to fit the measured data.

**Figure 4:**
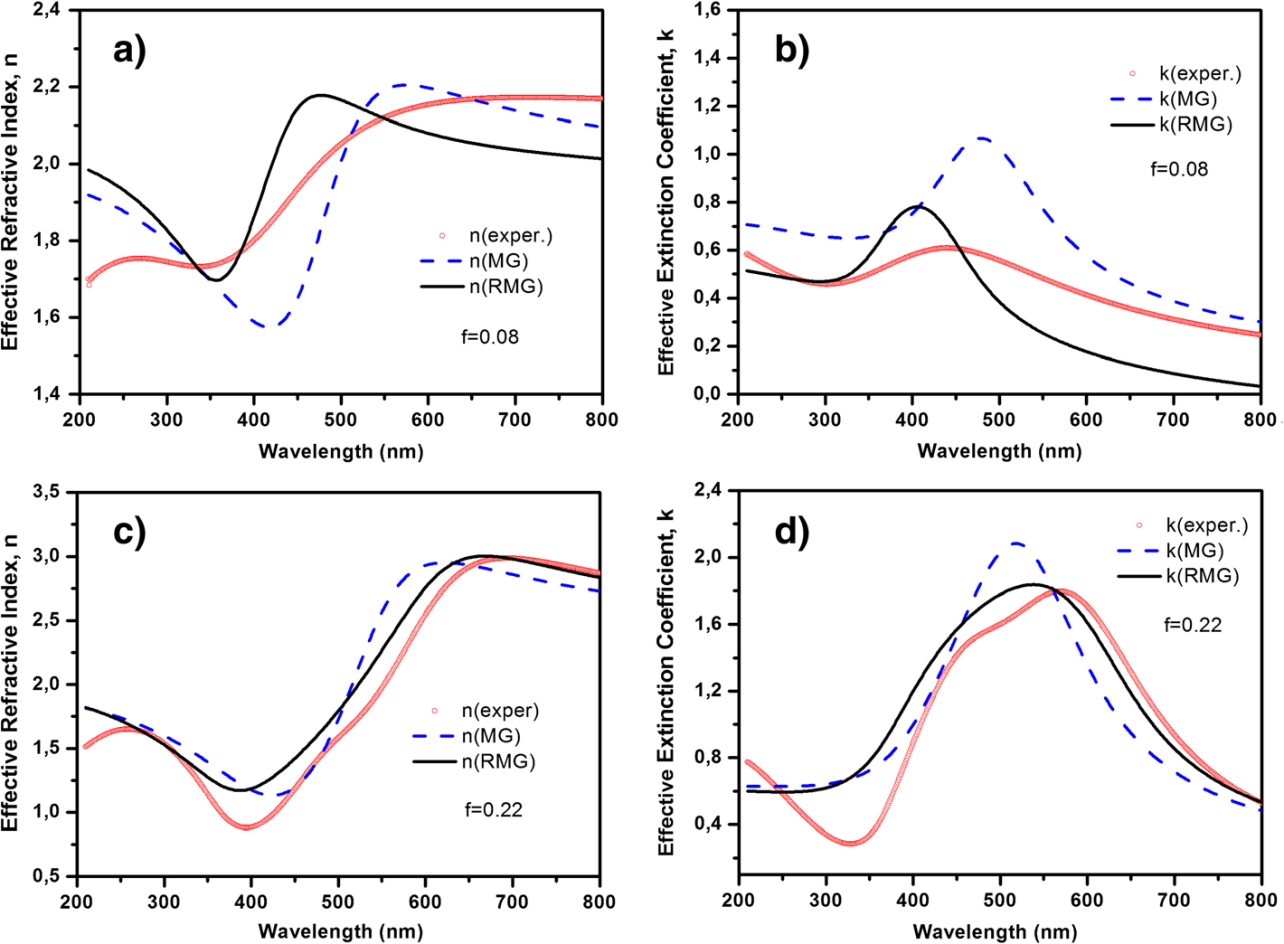
**Effective properties of the DLC:Ag films.** Effective refractive indices of films containing 8 **(a)** and 22 at.% **(c)** of Ag; effective extinction coefficients of films containing 8 **(b)** and 22 at.% **(d)** of Ag. Experimental curves are presented by circle. The effective properties were calculated by MG effective medium theory (dashed line), RMG effective medium theory (solid lines).

To check the proposed model, the effective refractive indices of DLC:Ag films were calculated by classical MG and RMG theories as well as compared with the experimental results for the films with filling fraction 0.08 and 0.22 (see Figure 4). The experimental effective refractive indices of the DLC:Ag films were obtained from regression analysis of spectroscopic ellipsometry data. Spectroscopic ellipsometry data were analyzed using layer model, which consisted of bulk SiO_2_ and layer of DLC with embedded Ag nanoparticles. In order to model the spectral dependence of ellipsometric parameters, Cody-Lorentz and two Lorentz oscillators were taken into account. Cody-Lorentz function determines optical dispersion of DLC, meanwhile Lorentz oscillators correspond to plasmons of the Ag nanoparticles. The calculation results were obtained for the nanoparticles that are spherical in shape. The average radius of nanoparticles for the sample with filling fraction 0.08 was 8 nm, and for the sample with filling fraction 0.22, it was 10 nm, according to the size distributions (see Figure 3). RMG had better fitting with the experimental results than the MG theory, as a result that dipole-dipole interactions the between nanoparticles was taken into account. Thus, all our further results were obtained using the RMG approximation.

**Figure 5:**
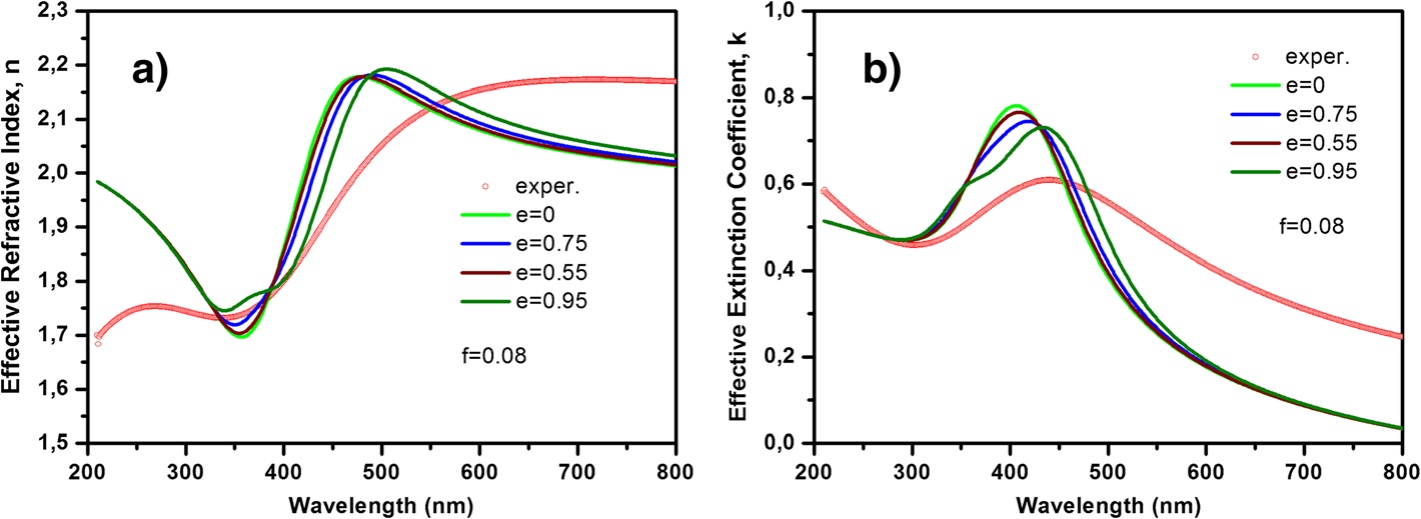
**Effective properties of DLC:Ag films containing nanoparticle of spherical shape.** Different eccentricity as indicated: **(a)** effective refractive index, **(b)** effective extinction coefficient (containing 8 at.% of Ag, averaged nanoparticles radius is 8 nm).

**Figure 6:**
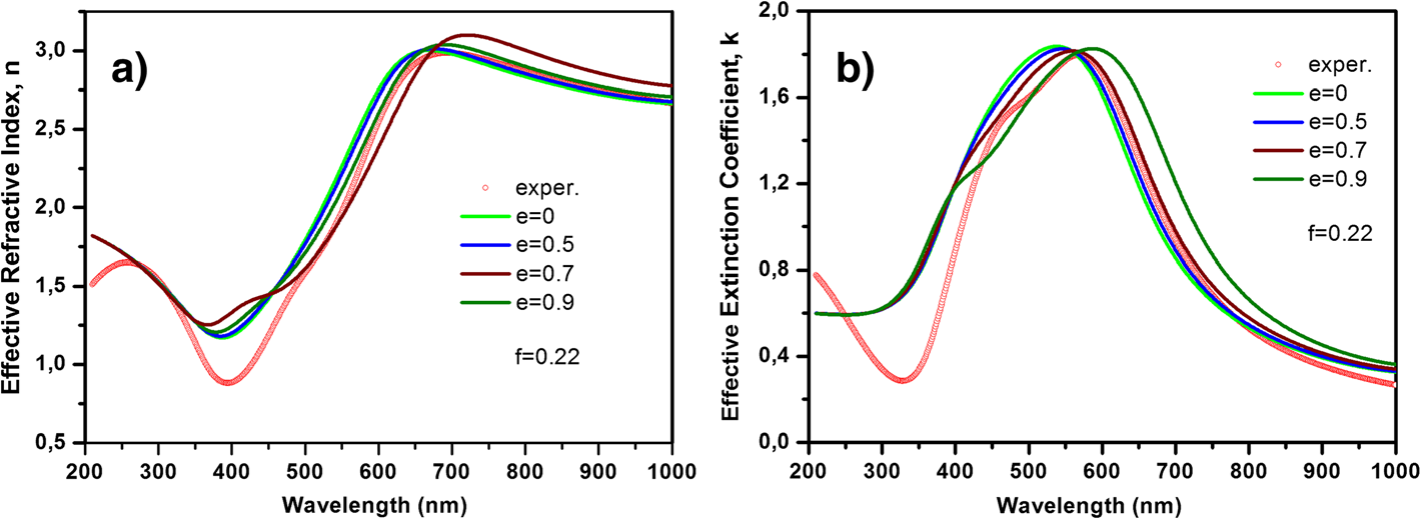
**Effect of the shape of the nanoparticles on nanocomposite film containing 22 at.% of Ag.** Effective properties of DLC:Ag films containing nanoparticle of spherical shape with different eccentricity as indicated: **(a)** effective refractive index**, (b)** effective extinction coefficient (containing 22 at.% of Ag, nanoparticles radius is 10 nm).

The experimental results show that incorporated nanoparticles in DLC matrix do not have ideal spherical shape (see Figure 1). To study influence of the nonsphericity on the effective properties of nanocomposite film, in the present calculations, we have used the Equation 1 for the effective dielectric permittivity taking into account expressions for depolarization coefficients (Equation 5). Figure 5 demonstrates the effect of the shape of the nanoparticles on the effective properties of nanocomposite film containing 8 at.% of Ag, and Figure 6 demonstrates the effect of the shape of the nanoparticles on the effective properties nanocomposite film containing 22 at.% of Ag. It is clearly seen that for bigger eccentricity, there are both the shift and the deformation of the curves. As a result, the curves will be shifted and broader peak of plasmon resonance will be detected. Therefore, the shape of the nanoparticles must be taken into account for modeling of the actual nanocomposites.

**Figure 7:**
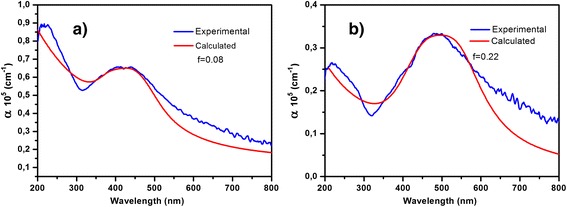
**Experimental and calculated absorption spectra (a,b).** DLC film containing Ag nanoparticles of spherical shape with different filling fractions as indicated.

Now, let us consider absorption of the composite thin films with filling fraction 0.08 and 0.22 (they correspond to the silver atomic concentration 22 and 8 at.%, respectively). Comparison of the experimental results with the calculation results for some eccentricities values (*e*) of the nanoparticles allows us to choose theoretical curve with good experimental fitting. Theoretical curves of the effective optical parameters have the best fitting when eccentricity 0.75 was chosen for the film with silver atomic concentration 8% and eccentricity 0.7 for the film containing 22 at.% of Ag (see Figures 5 and 6). Therefore, absorption spectra of these nanocomposites were calculated according to Equation 6 taking into account not only the dipole-dipole interactions between the nanoparticles but their nonsphericity as well, that is presented by eccentricity *e*. The experimental and theoretical absorption spectra are presented in Figure 7.

Not ideal fitting of the experimental curves by the theoretical ones can be explained by some reasons: firstly, we assume that all our particles have oblate shape that is not evident exactly; secondly, we used the averaged radius; and last, as it was shown in our previous work [9], peak of surface plasmon is very sensitive to material of the host matrix. In our case, DLC properties are very sensitive to the gas composition and other technological conditions during deposition [30], thus the small change of the DLC refractive index results in the shift of peak position of plasmon resonance of the nanocomposite. In general, our results demonstrate that shape nanoparticles and interaction between them have significant influence on optical absorption spectra DLC:Ag films.

## Conclusions

DLC:Ag films consisting of nanosized metal particles embedded in a diamond carbon matrix have been deposited on Si substrates using reactive magnetron sputtering. The nanocomposite structure and composition were observed by EDS profiling, TEM, and AFM. The experimental data (size and shape of nanoparticles) are strongly supported by the modeling results. The renormalized Maxwell-Garnett approximation was used to fit the experimental spectrum, using effective of optical parameters for the calculation. We can conclude that such theory is suitable to describe the absorption of silver nanoparticles with different deformations and radii, incorporated in diamond-like carbon films, and effective properties of the nanocomposite can be used for predicting their optical response. The results indicate that the broadening of absorption spectra are caused by the break of the spherical symmetry of the nanoparticles and interaction between them. Experimental study by means of TEM and AFM as well as modeling revealed that atomic force microscopy can be used to measure the size of the silver nanoclusters embedded into DLC:Ag film.
